# Out of India, thrice: diversification of Asian forest scorpions reveals three colonizations of Southeast Asia

**DOI:** 10.1038/s41598-020-78183-8

**Published:** 2020-12-18

**Authors:** Stephanie F. Loria, Lorenzo Prendini

**Affiliations:** 1 Richard Gilder Graduate School, American Museum of Natural History, Central Park West at 79th St., New York, NY 10024-5192 USA; 2 Scorpion Systematics Research Group, Division of Invertebrate Zoology, American Museum of Natural History, Central Park West at 79th St., New York, NY 10024-5192 USA

**Keywords:** Zoology, Phylogenetics, Speciation, Evolution, Evolutionary genetics

## Abstract

The ‘Out of India’ hypothesis is often invoked to explain patterns of distribution among Southeast Asian taxa. According to this hypothesis, Southeast Asian taxa originated in Gondwana, diverged from their Gondwanan relatives when the Indian subcontinent rifted from Gondwana in the Late Jurassic, and colonized Southeast Asia when it collided with Eurasia in the early Cenozoic. A growing body of evidence suggests these events were far more complex than previously understood, however. The first quantitative reconstruction of the biogeography of Asian forest scorpions (Scorpionidae Latreille, 1802: Heterometrinae Simon, 1879) is presented here. Divergence time estimation, ancestral range estimation, and diversification analyses are used to determine the origins, dispersal and diversification patterns of these scorpions, providing a timeline for their biogeographical history that can be summarized into four major events. (1) Heterometrinae diverged from other Scorpionidae on the African continent after the Indian subcontinent became separated in the Cretaceous. (2) Environmental stresses during the Cretaceous–Tertiary (KT) mass extinction caused range contraction, restricting one clade of Heterometrinae to refugia in southern India (the Western Ghats) and Sri Lanka (the Central Highlands). (3) Heterometrinae dispersed to Southeast Asia three times during India’s collision with Eurasia, the first dispersal event occurring as the Indian subcontinent brushed up against the western side of Sumatra, and the other two events occurring as India moved closer to Eurasia. (4) Indian Heterometrinae, confined to southern India and Sri Lanka during the KT mass extinction, recolonized the Deccan Plateau and northern India, diversifying into new, more arid habitats after environmental conditions stabilized. These hypotheses, which are congruent with the geological literature and biogeographical analyses of other taxa from South and Southeast Asia, contribute to an improved understanding of the dispersal and diversification patterns of taxa in this biodiverse and geologically complex region.

## Introduction

The ‘Out of India’ hypothesis is often invoked to explain patterns of distribution among Southeast Asian taxa^1–10^. According to this hypothesis, the ancestors of Southeast Asian taxa originated in Gondwana, diverged from their Gondwanan relatives when the Indian subcontinent (including present-day India, Bangladesh, Nepal, Pakistan and Sri Lanka) rifted from Gondwana, rafted on the subcontinent, and arrived at Southeast Asia during or after it collided with Eurasia in the early Cenozoic. Support for the ‘Out of India’ hypothesis crosses taxonomic boundaries. Research on plants^2,11,12^, fish^13–15^, amphibians^1,3,4,16–20^, birds^21^, lizards^10,22^, and arthropods^5,8,9,23^ suggest an Indian origin for many Southeast Asian taxa. The Indian-Eurasian collision also resulted in colonization of the Indian subcontinent by Eurasian taxa, the so-called ‘Into India’ hypothesis, although there are fewer supporting examples^24,25^.

The path of the Indian subcontinent on its northward drift, and the timing of its collision with Eurasia have been extensively debated^26–42^. Many geologists and biogeographers favor an early collision date, occurring 55–50 Ma at the Paleocene–Eocene boundary^30,32,33,40–42^, based largely on geological data including seafloor spreading, tectonic plate movement rates, biostratigraphy, and paleontological data^41,42^. This ‘traditional’ hypothesis fails to explain why effects of the Indian-Eurasian collision do not appear until 30 Ma, 20 million years after the collision commenced^26^.

More recent hypotheses account for this discrepancy by suggesting that India experienced several periods of contact with Eurasia, the initial contact occurring as early as 55 Ma, and the final India-Eurasian collision occurring between 35–20 Ma^26,27,38^. For example, Li et al.^19^ proposed that rhacophorid frogs first colonized Southeast Asia from India during the Eocene (57–46 Ma). Faunal exchange between India and Southeast Asia ceased during the Middle Eocene but resumed between the Oligocene and Middle Miocene (ca. 34–12 Ma). Although several studies suggest faunal exchange between India and Southeast Asia may have occurred more than once, opinions differ as to how the initial contact occurred. According to Aitchison et al.^26^ and Ali and Aitchison^27^^,^ Greater India brushed up against Sumatra and collided with an intra-oceanic island arc, the Dazhuqu Arc, ca. 55 Ma in the Neo-Tethys Ocean. India then moved closer to Eurasia until its final collision, roughly 34 Ma^26,27^. Two alternative hypotheses were proposed for the location of India between 55–35 Ma, according to which India was situated either very close to Southeast Asia^28^ or slightly further away^29^. The hypothesis of Ali and Aitchison^27^ was supported by a biogeographical analysis of freshwater crabs, which suggested that dispersal from India to Southeast Asia occurred during the Middle Eocene as India brushed up against Southeast Asia before the final collision^9^. In contrast, Van Hinsbergen et al.^38^ proposed that initial contact between India and Eurasia occurred when a microcontinent, the Tibetan Himalayan landmass, rifted from northern India and collided with Eurasia approximately 50 Ma. The rest of the Indian subcontinent was isolated following this initial rift until colliding much later (25–20 Ma). Aitchison and Ali^35^ refuted the hypothesis of Van Hinsbergen et al.^38^, suggesting the geological data were misinterpreted.

The hypothesis of Gondwanan taxa rafting on the Indian subcontinent is more complex than the number of colonizations of Southeast Asia, however. Approximately 65 Ma, the Deccan Traps, situated in the center of the subcontinent, underwent extreme volcanic activity, coinciding with the meteorite collision in the Chicxulub Crater, Mexico, and lasting less than a million years^43–55^. These volcanic eruptions, combined with effects of the Chicxulub impact, resulted in drastic environmental changes across the globe and are thought to have caused the Cretaceous–Tertiary (KT) mass extinction^43,45,46,48,51–55^. The Western Ghats of India and the Central Highlands of Sri Lanka have been posited as potential refugia during this period of Deccan volcanism^56–58^. Bossuyt et al.^17^ suggested frog lineages survived in southern India and Sri Lanka during the intense volcanism and subsequently dispersed across Eurasia after India’s collision. Numerous studies identified close relationships between taxa inhabiting the Western Ghats, southern India, and Sri Lanka^58–63^. Conti et al.^2^ proposed that the angiosperm family Crypteroniaceae de Candolle, 1868 reached Southeast Asia from the Indian subcontinent and explained its absence from India as the result of extinction caused by climatic changes (e.g., associated with volcanism) at the end of the Cretaceous and in the early Tertiary but survived in refugia, including Sri Lanka.

**Figure 1:**
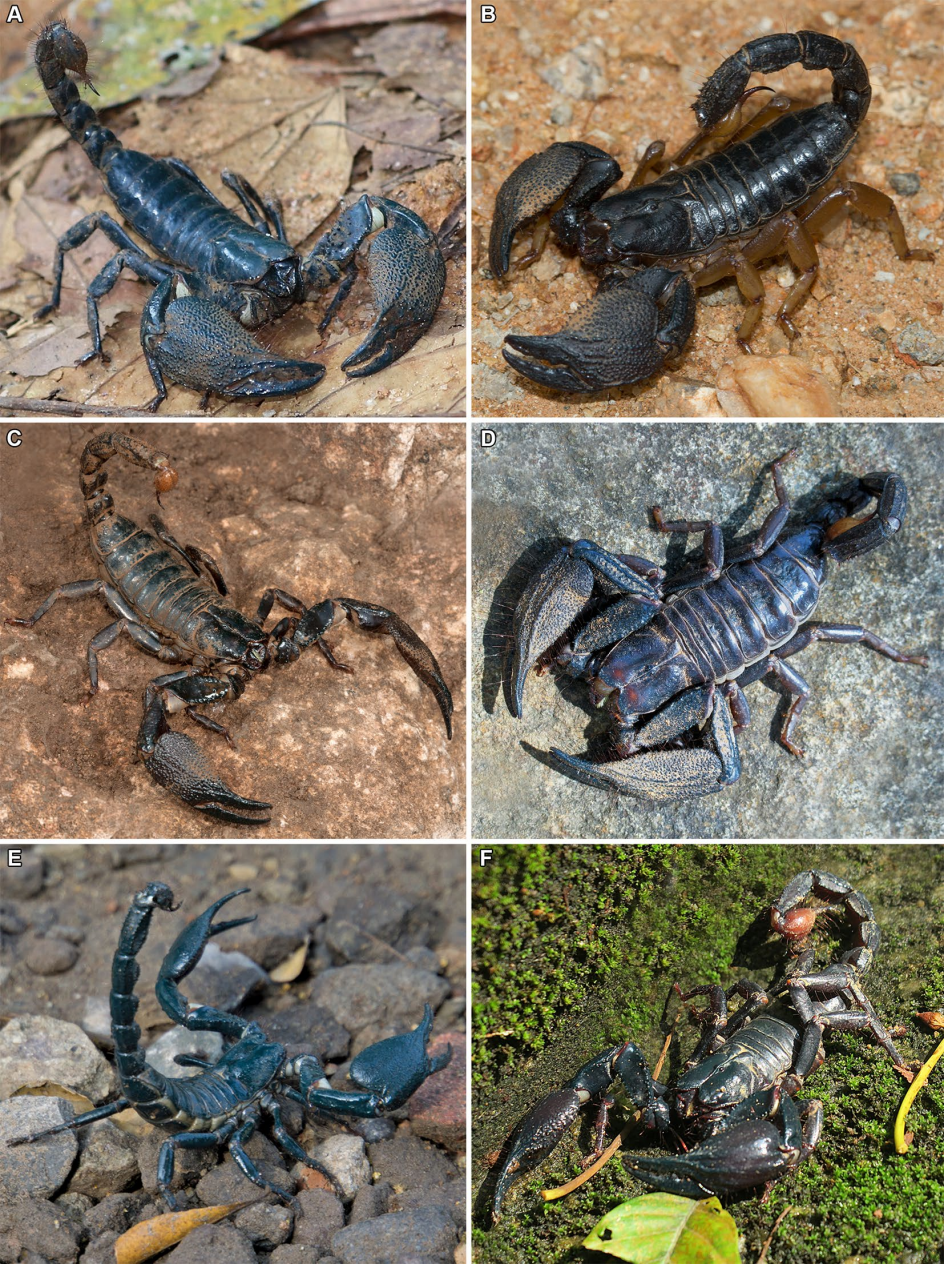
Representative species of Asian forest scorpions (Scorpionidae: Heterometrinae). (**A**) *Srilankametrus serratus* (Pocock, 1900), ♂, Sinharaja Forest Reserve, Sabaragamuwa Province, Sri Lanka. (**B**) *Chersonesometrus madraspatensis* (Pocock, 1900), ♀, Gautala Wildlife Sanctuary, Maharashtra, India. (**C**) *Deccanometrus phipsoni* (Pocock, 1893), ♂, Achanakmar Tiger Reserve, Chhattisgarh, India. (**D**) *Chersonesometrus tristis* (Henderson, 1919), ♂, Kaigal Falls, Andhra Pradesh, India. (**E**) *Javanimetrus cyaneus* (C.L. Koch, 1836), ♂, Gunung Kidul, Special Region of Yogyakarta, Indonesia. (**F**) *Heterometrus petersii* (Thorell, 1876), ♂, Central Water Catchment, Singapore.

The scorpion family Scorpionidae Latreille, 1802 is distributed across most of Africa (except the Sahara), the Middle East, the Indian subcontinent, and Southeast Asia^5,23,64^. Asian members of the family, i.e., Heterometrinae Simon, 1879, also known as Asian forest scorpions (Fig. 1), are distributed from Pakistan in the northwest, across India and Sri Lanka to Southeast Asia, reaching Wallace’s Line, between Borneo and Sulawesi, in the east^5^. The subfamily previously included only a single genus, *Heterometrus* Ehrenberg, 1828, with five subgenera recognized by some authors^23,65,66^, but a recent revision identified 41 species and seven reciprocally monophyletic and morphologically distinct clades^67^, redefined as genera: *Chersonesometrus* Couzijn, 1978; *Deccanometrus* Prendini & Loria ^67^; *Gigantometrus* Couzijn, 1978; *Heterometrus*; *Javanimetrus* Couzijn, 1981; *Sahyadrimetrus* Prendini & Loria^67^; *Srilankametrus* Couzijn, 1981. These fossorial scorpions construct burrows in primary and secondary rainforests, dry deciduous forests, savanna and scrubland^68,69^.

**Figure 2:**
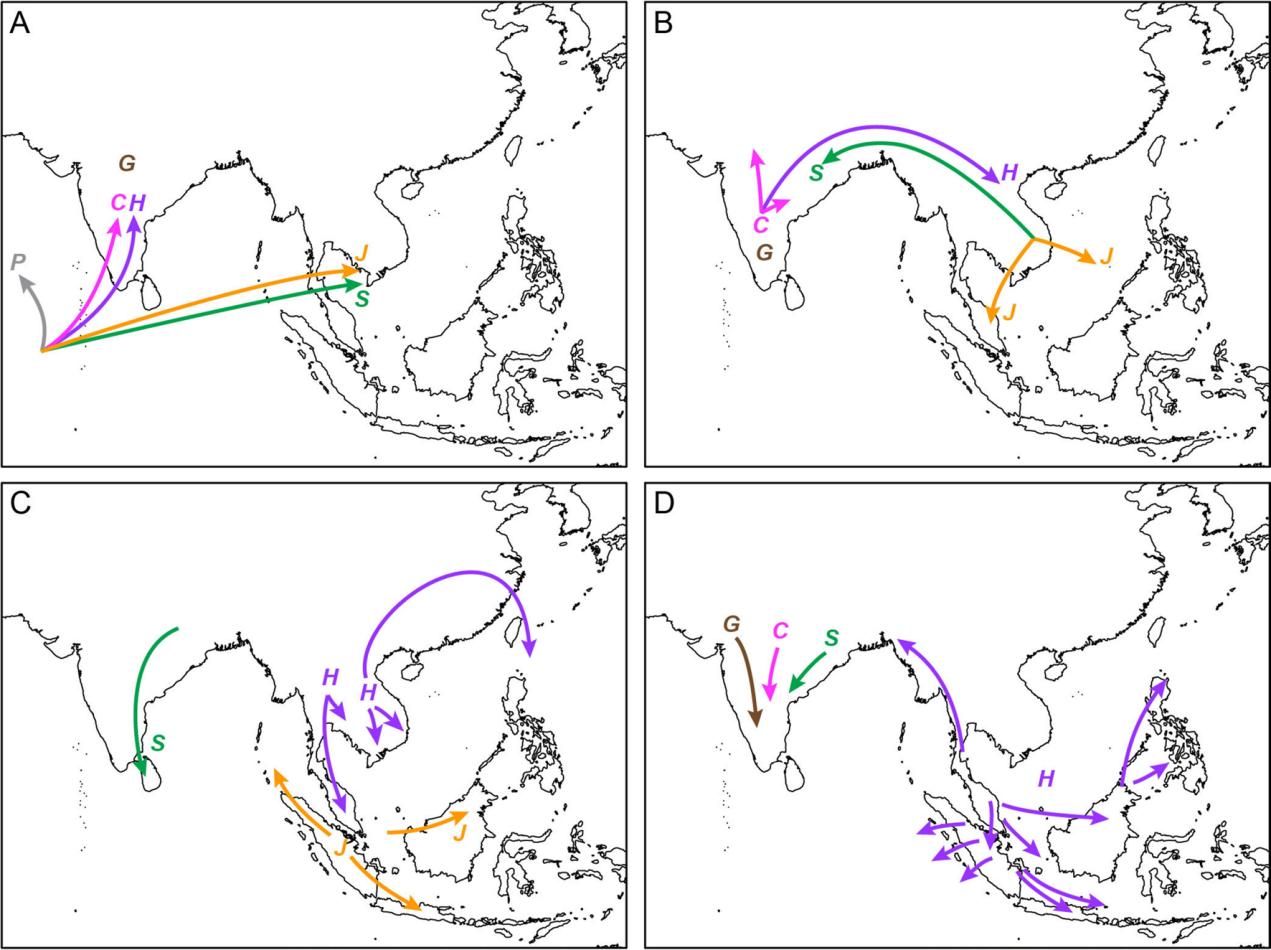
Couzijn’s^23^ hypothesis for the evolution and diversification of the Asian forest scorpions (Scorpionidae: Heterometrinae). (**A**) Gondwana origins and divergence: Heterometrinae diverge from *Pandinus* (Pandininae) (*P*) on Gondwana; the Indian subcontinent rifts from Gondwana, carrying *Gigantometrus* (*G*) and the ancestor of (*Chersonesometrus* + *Heterometrus*); the Indochinese landblock rifts from Gondwana, carrying the ancestor of (*Javanimetrus* + *Srilankametrus*)*.* (**B**) Dispersal across the Assam gateway: *Heterometrus* (*H*)  disperses east to Southeast Asia; *Srilankametrus* (*S*) disperses west to India; *Javanimetrus* (*J*) disperses south and southeast to Borneo, the Philippines and the Malay Peninsula; *Chersonesometrus* (*C*) disperses north across the Indian subcontinent. (**C**) Tertiary dispersal: *Srilankametrus* disperses south to Sri Lanka; *Javanimetrus* disperses northwest to Sumatra and the Nicobar Islands, and east to Java and Borneo; *Heterometrus* disperses south to Indochina and the Malay Peninsula, and north to the Philippines. (**D**) Pleistocene dispersal: *Heterometrus* disperses more widely across its range; the Indian taxa disperse south due to glaciation in the Himalayas.

The biogeography of Heterometrinae has received some attention^5,23,64,70–73^. According to Couzijn^23^, the ancestor of Heterometrinae and Pandininae Thorell, 1876 originated in eastern Gondwana during the Triassic (Fig. 2). During the breakup of Gondwana, different lineages of Heterometrinae diverged in isolation on separate fragments of the former supercontinent. *Chersonesometrus* and *Gigantometrus* originated on the Indian plate and were carried north as it moved towards the rest of Asia while the ancestor of *Javanimetrus* was carried north on the Indochinese shelf complex^74^, which ultimately collided with Southeast Asia (Fig. 2A). After these landmasses arrived at their present locations, by the end of the Cretaceous, an exchange of taxa occurred via the Assam gateway (Fig. 2B). The ancestor of *Heterometrus*, endemic to Southeast Asia, originated in India, where it shares a common ancestor with the Indian *Chersonesometrus*, and dispersed east whereas the ancestor of *Srilankametrus*, endemic to southern India and Sri Lanka, originated in Southeast Asia, where it shares a common ancestor with the Southeast Asian *Javanimetrus*, and dispersed west^73^. During the Tertiary, an island chain known as the Luzon Track, which includes Taiwan, the Philippines and Borneo, formed in the Sulu Basin, allowing *Heterometrus* to disperse to these islands from northern Indochina while other *Heterometrus* species dispersed south to the Malay Peninsula, Sumatra, Borneo, and Java (Fig. 2C). *Srilankametrus* meanwhile dispersed from India to Sri Lanka while *Javanimetrus* dispersed from Sumatra northwest to the Nicobar Islands and southeast to Java (Fig. 2C). During the Pleistocene, the colder and drier climate caused by glaciation in the Himalayas forced some Indian Heterometrinae to disperse to southern India and Sri Lanka, while sea level changes allowed species of *Heterometrus* to expand their ranges in Southeast Asia (Fig. 2D).

Couzijn’s^23^ biogeographical hypothesis has several implications. The basal phylogenetic position of *Gigantometrus*, which inhabits southern India and Sri Lanka, suggests Asian scorpionids originated in the southern part of the Indian subcontinent. Couzijn’s^23^ ‘Out of India’ hypothesis for *Heterometrus* and ‘Into India’ hypothesis for *Srilankametrus* imply two dispersal events and two extinction events, as *Heterometrus* are absent from the Indian subcontinent and *Srilankametrus* are absent from Southeast Asia. An ‘Out of India’ hypothesis for Heterometrinae was also supported by Prendini’s^64^ phylogeny of superfamily Scorpionoidea Latreille, 1802, based on morphological data for exemplar species. Prendini^64^ recovered Scorpionidae as monophyletic, with the Asian *Heterometrus* sister to the African *Pandinus* Thorell, 1876, implying that Asian scorpionids diverged from their African relatives when India separated from Africa. Prendini et al.^5^ corroborated this hypothesis with a phylogeny of Scorpionidae based on morphology and DNA sequence data from five gene loci, for a larger taxon sample, which also recovered two biogeographically distinct clades among the Asian scorpionid exemplars, representing species from the Indian subcontinent and Southeast Asia, respectively.

The first quantitative reconstruction of the biogeography of Asian forest scorpions (Heterometrinae) is presented here. Divergence time estimation, ancestral range estimation, and diversification analyses are used to determine the origins, dispersal and diversification patterns of the subfamily, and place that in the context of modern understanding of the biogeographical history of South and Southeast Asia.

## Results

### Divergence-time estimate analysis

The divergence-time estimation analysis in BEAST produced a fully dichotomous phylogeny. Posterior probability values were generally high and the monophyly of most genera well supported (≥ 0.99; Fig. 4). Relationships among the genera were also well supported (≥ 0.92) and congruent with the phylogeny based on a more extensive taxon sample (Supplementary Figure).

### Ancestral range estimation and phylogenetic diversity

**Figure 3:**
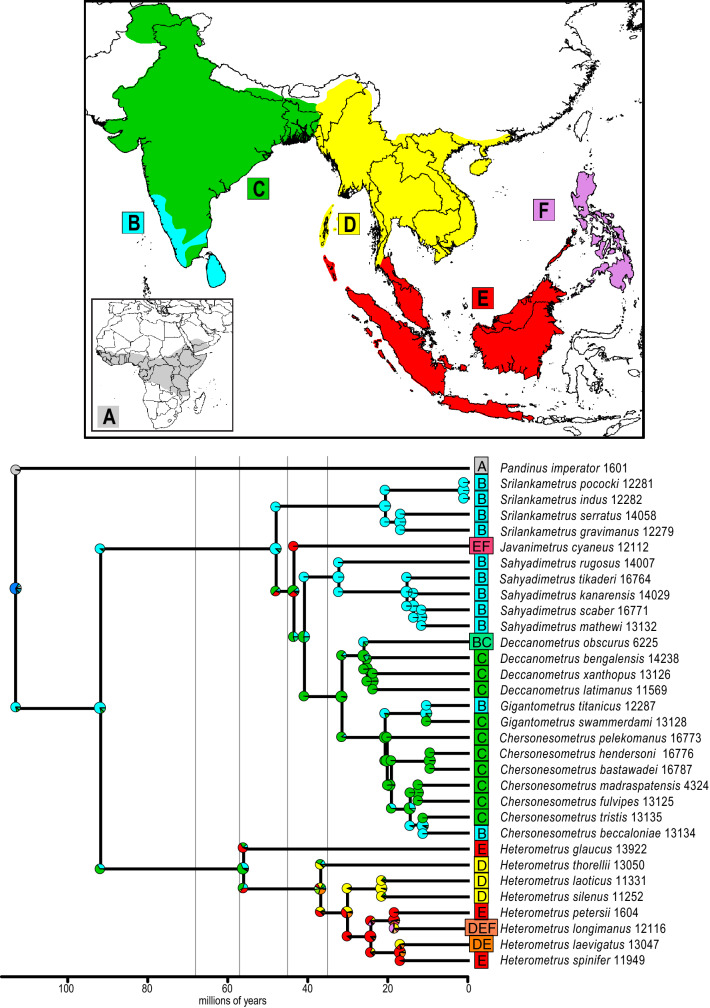
Estimation of ancestral ranges of the Asian forest scorpions (Scorpionidae: Heterometrinae) in six biogeographical regions: Africa (**A**); Western Ghats and Sri Lanka (**B**); Greater Indian Subcontinent (**C**); Indochina (**D**); Sundaland (**E**); Philippines (**F**). Lines on phylogeny indicate periods in the time-stratified analysis: 0–35 Ma, 35–45 Ma, 45–57 Ma, 57–68 Ma, 68–120 Ma. Map produced using ArcGIS v. 10.1 with ESRI (http://www.esri.com) *World Countries* layer, DeLorme Publishing Company, Inc. (2013).

Comparison of the log likelihood, AIC and AICc values of several models identified the DEC + *j* model as the most appropriate for the dataset (Table 3). Based on this model, the following ranges were recovered as ancestral for each genus: Western Ghats and Sri Lanka for *Gigantometrus*, *Sahyadrimetrus* and *Srilankametrus* and the Greater Indian Subcontinent for *Chersonesometrus*, *Deccanometrus*, *Heterometrus* and *Javanimetrus* (Table 2; Fig. 3). The Western Ghats and Sri Lanka had the highest phylogenetic diversity (PD) among the six biogeographical areas, whereas the Philippines had the lowest (Table 4). *Heterometrus glaucus* (Thorell, 1876) had the highest evolutionary distinctiveness (ED) among the species (Fig. 5).

### Diversification analyses

Net diversification rates were as follows: 0.023 for high extinction rate (ε = 0.9) and high number of missing species (*n* = 50); 0.016 for high extinction rate (ε = 0.9) and low number of missing species (*n* = 5); 0.040 for low extinction rate (ε = 0) and high number of missing species (*n* = 50); 0.031 for low extinction rate (ε = 0) and low number of missing species (*n* = 5). Comparison of AIC values for seven models identified the two-rate Yule model as the best fit for the dataset (Table 5). The gamma statistic was negative (γ = − 2.08) and statistically significant (*p* = 0.019) suggesting most branching occurred early in the phylogeny. A Monte Carlo Constant Rates (MCCR) test indicated that the gamma statistic was affected by incomplete sampling when at least 28% of the taxa were missing (*p* > 0.05) (Table 6). A phylorate plot produced using BAMM supported fast speciation rates, occurring early in the phylogeny, and calculation of rate shifts indicated one distinct rate shift occurred (Fig. 5). An LTT plot also supported a declining speciation rate over time (Fig. 5).

## Discussion

### Asian Scorpionidae originate on Indian subcontinent during Cretaceous

The phylogenetic analyses presented here confirm previous studies^5,23,64^ in supporting the monophyly of Asian Heterometrinae and their sister group relationship with African Pandininae, represented in the analysis by *Pandinus*. This relationship implies Scorpionidae originated on the African continent and Heterometrinae diverged from their African relatives following the separation of West Gondwana (comprising Africa and South America) from East Gondwana (comprising the Indian subcontinent, Madagascar, Seychelles, Australia, and Antarctica). The East–West Gondwana rift has been invoked to explain biogeographical patterns among many taxa^75,76^ but the timing of the complete isolation of the Indian subcontinent from Africa has been debated. Prior to the East–West Gondwana rift, India was connected to Africa via Madagascar and Antarctica^77^, and it is likely Scorpionidae spread across this area. Land connections between India and Africa began to sever ca. 175 Ma^27,29,78^. Rifting began in the north, by opening of the Somali Basin between India-Madagascar and present-day Somalia^79^, and shortly thereafter in the south, between Antarctica and present-day Mozambique, by seafloor spreading in the Mozambique Basin and opening of the Weddell Sea^79,80^. Antarctica was completely separated from West Gondwana by 135 Ma^78,81^, and seafloor spreading between Madagascar and Africa had ceased ca. 120 Ma^75,82,83^, ending the rift between West and East Gondwana. However, despite India being physically separated from West Gondwana by this time, mounting evidence suggests India’s biota was not completely isolated from West Gondwana but rather that connections between India and Africa continued well into the Late Cretaceous, possibly even into the early Paleocene^27,84–89^. For example, several Late Cretaceous–early Paleocene fossil tetrapods, discovered in India, reveal close affinities with African relatives, suggesting connectivity between the two landmasses until ca. 65 Ma^88^. A divergence time estimation of two extant frog clades, based on DNA and calibrated using fossils, also supports a link between India and Africa until roughly 85 Ma in the Late Cretaceous^86^.

Several geological explanations have been proposed to explain connections between the faunas of Africa and India in the Late Cretaceous. Chatterjee and Scotese^84^ proposed a land bridge, connecting northwestern India to eastern Arabia, known as ‘Greater Somalia’. However, this hypothesis was refuted by geological data^87,90^, which demonstrated that, by the Late Triassic–Early Jurassic, Greater Somalia had already broken into two plates, situated far from the Indian subcontinent. Instead, an alternative explanation was proposed, the ‘Oman-Kohistan-Ladakh Island Arc’ hypothesis, which states that an island chain connected the Indian subcontinent with northern Africa between 80 and 65 Ma, or possibly even later^87,91–93^. A major revision of the plate tectonic and paleogeographical model of the Indian subcontinent^27^ criticized many plate tectonic models of India proposed by biogeographers for failing to consider geological evidence. Ali and Aitchison^27^ nevertheless supported the notion that the Indian subcontinent was not totally isolated after the East–West Gondwana rift. Their model suggested biotic exchange could have occurred between India and Antarctica-Australia via the Kerguelen Plateau until 90 Ma, and between India and Africa until 85 Ma with a more limited connection via the Madagascar-Providence Bank-Amirante Ridge-Seychelles block^27^. According to the model, India had reached its maximum isolation by 68 Ma, making it unlikely that faunal exchange would have occurred during this time, although limited connections to Africa may have existed.

**Figure 4:**
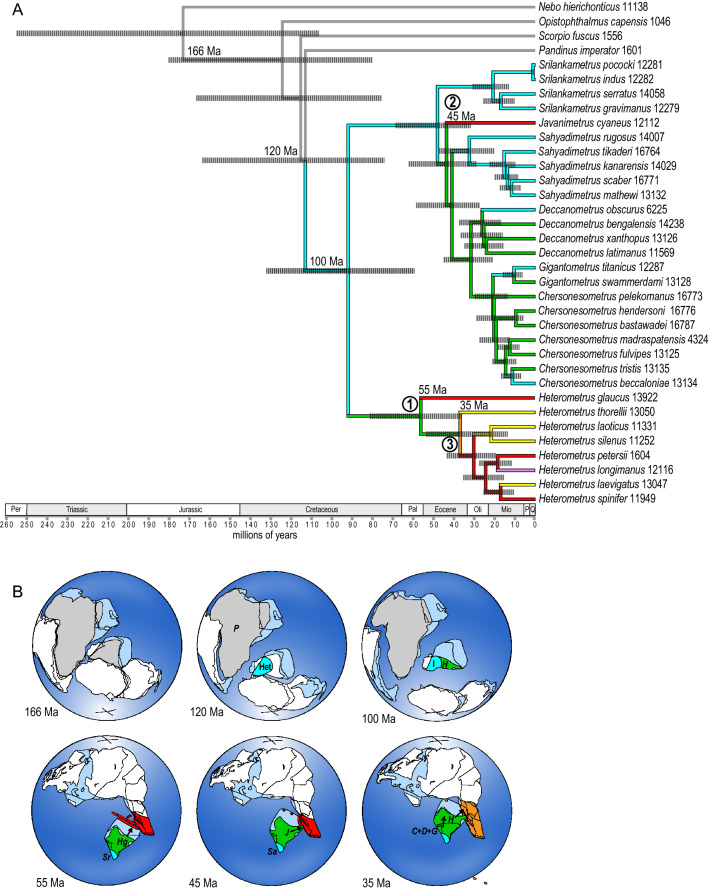
Estimation of divergence times (**A**) in evolution of the Asian forest scorpions (Scorpionidae: Heterometrinae) with paleogeographical reconstructions (**B**) indicating tectonic positions of India at various geological time periods, following Ali and Aitchison^27^ and Acton^28^. Continental outlines indicate past and present plate boundaries and colored regions indicate ancestral ranges for clades in the phylogeny (**A**). ‘X’ marks the south (166 Ma, 120 Ma, 100 Ma) and north poles (55 Ma, 45 Ma, 35 Ma). Only relevant landmasses are modeled^27^ in the 55 Ma, 45 Ma and 35 Ma paleogeographical reconstructions. Geological time periods on the phylogeny include Permian (Per), Triassic, Jurassic, Cretaceous, Paleocene (Pal), Eocene, Oligocene (Oli), Miocene (Mio), Pliocene (P) and Quaternary (Q). Circled numbers on phylogeny denote three dispersal events (55 Ma, 45 Ma and 35 Ma) of Heterometrinae from India to Southeast Asia. Ages (in millions of years) plotted on phylogeny refer to paleomap ages not node ages. Timeline for the origins, dispersal and diversification of Heterometrinae (**B**) as follows. 166 Ma: Africa begins to rift from India. 120 Ma: Heterometrinae (Het) diverge from sister group, the African Pandininae (P) as India becomes isolated from Africa. 100 Ma: Heterometrinae splits into two clades: one confined to southern India and Sri Lanka (I) and the ancestor of *Heterometrus* (*H*), widely distributed across the Indian subcontinent. 55 Ma: The ancestor of *Heterometrus glaucus* (*Hg*) disperses to Sundaland as the Indian subcontinent brushes up against the western coast of Sumatra; *Srilankametrus* (*Sr*) diverges and later diversifies across the Western Ghats and Sri Lanka. 45 Ma: The ancestor of *Javanimetrus* (*J*) disperses to Sundaland; *Sahyadrimetrus* (*Sa*) diverges and later diversifies across the Western Ghats and Sri Lanka. 35 Ma: The ancestor of a clade comprising most of the extant species of *Heterometrus* disperses to Indochina and Sundaland; the ancestor of the clade comprising *Chersonesometrus* (*C*), *Deccanometrus* (*D*) and *Gigantometrus* (*G*) disperses northwards across the Indian subcontinent.

According to the molecular clock calibrations of the present study, Asian scorpionids diverged from their African relatives ca. 113 Ma, supporting hypotheses of earlier biological connectivity between Africa and the Indian subcontinent^1^. Paleogeographical reconstructions of India for this period^27^ indicate the landmass comprising India and Madagascar drifting northwards along the eastern side of southeastern Africa (Fig. 4). Prior to 113 Ma, rafting between Africa and India may have been possible via the precursors of Madagascar or the Seychelles, allowing gene flow between Asian scorpionids and their African relatives, until the distance between these landmasses became too great and rafting was impossible. Rafting has been proposed for other scorpion taxa^94–96^ and, given that some Heterometrinae have been found in rotting logs and appear able to withstand habitat disturbance^67^, this means of dispersal is plausible. More paleontological and phylogenetic studies of African and Indian taxa, as well as geological evidence, are needed to refine the timing and mechanism of divergence between African and Asian scorpionids, however.

### Range contraction of Indian Heterometrinae around KT boundary

The phylogeny suggests that, after diverging from their African relatives, the Asian scorpionids diverged into two major clades. One clade includes *Chersonesometrus*, *Deccanometrus*, *Gigantometrus* and *Sahyadrimetrus* from the Indian subcontinent, and the monotypic genus, *Javanimetrus*, known from the Nicobar Islands, peninsular Thailand, and several Indo-Pacific islands. The other comprises *Heterometrus*, widely distributed throughout Southeast Asia including the Andaman and Nicobar Islands, Indochina, the Thai-Malay Peninsula and many Indo-Pacific islands to the west of Wallace’s Line. The diversification time and ancestral range analyses estimate this divergence occurred approximately 92 Ma in the Late Cretaceous.

At the end of the Cretaceous, starting 67.4 Ma, the Deccan Traps erupted, and India experienced a period of intense volcanism that occurred in three phases, interrupted by nonvolcanic phases, over a short timespan lasting less than 1 million years^51^. During these eruptions, between 1.2 and 1.5 km^3^ of lava extruded across India, especially the present-day states of Gujarat, Madhya Pradesh, and Maharashtra, with the longest lava flow known in the world reaching as far south as the Krishna-Godavari Basin in Andhra Pradesh^49,51^. Large amounts of volcanic gases were also released with estimates of 15,000–35,000 Gt of carbon dioxide (CO_2_) and 6800–17,000 Gt of sulphur dioxide (SO_2_) entering the atmosphere during the most intense phase of volcanic eruption^47,51^. Despite the severity of this volcanic activity, the role of the Deccan eruptions in the KT mass extinction has been debated. Indian paleontological records reveal that many terrestrial organisms survived the eruptions^50^. Consequently, the meteorite impact at the end of the Late Cretaceous, which created the Chicxulub Crater off the Caribbean coast of Mexico, is favored as the primary cause for mass extinctions across the globe, and many studies argue that the effects of Deccan volcanism were only accessory to the environmental changes caused by the meteorite impact^45,53^. Some paleontological and geological data suggest the effects of Deccan volcanism were not insignificant, however, leading several researchers to argue that the Deccan eruptions were the primary cause of the global KT mass extinction^49,51,54,55^. For example, Schoene et al.^54^ used uranium-lead geochronology to demonstrate that the environmental conditions, which led to mass extinction, initiated during the main phase of eruptions, prior to the Chicxulub meteorite impact, suggesting volcanism was a major contributor to extinction. Palynological data from the Deccan region also indicate a shift in composition of the floral community after these eruptions. The flora was more diverse, and included an abundance of gymnosperms, before the eruptions, whereas angiosperms and pteridophytes dominated afterwards^48,50^. Examination of exposed marine beds on India demonstrate a major extinction of planktonic foraminifera with nearly all species disappearing after the last megaflow due to ocean acidification caused by volcanic gases^51,52^.

Although the contribution of the Deccan eruptions to the KT mass extinction, relative to the Chicxulub meteorite impact, remains unclear, several studies suggest diverse taxa were confined to the southern part of the Indian subcontinent, specifically the Western Ghats of India and the Central Highlands of Sri Lanka, at the end of the Late Cretaceous^1,2,17,57^. Consequently, these areas have been posited as potential refugia during the KT mass extinction. The results presented here support the hypothesis that the Western Ghats and Central Highlands of Sri Lanka were refugia and suggest Asian scorpionids experienced range contraction during the KT mass extinctions due to stresses caused by the Deccan eruptions, the Chicxulub meteorite impact, or both. The phylogeny and ancestral range estimation analyses support this hypothesis as the ancestor of the largely Indian clade, comprising *Chersonesometrus*, *Deccanometrus*, *Gigantometrus*, *Javanimetrus*, *Sahyadrimetrus* and *Srilankametrus*, was confined to the Western Ghats and Sri Lanka during this period. *Sahyadrimetrus* and *Srilankametrus*, endemic to the Western Ghats and Sri Lanka, are basal in the Indian clade, whereas *Chersonesometrus*, *Deccanometrus* and *Gigantometrus*, more widespread on the Indian subcontinent, are more distal. No new lineages of Heterometrinae arose between 92 and 56 Ma (Fig. 4).

### Three dispersal events follow Indian collision with Eurasia

The ‘Out of India’ hypothesis, according to which India acted as a raft carrying the ancestors of Asian taxa from Gondwana to Eurasia on its northward journey, has been invoked to explain the biogeographical history of many taxa, including Asian forest scorpions^1–10,23,73,97^. The present study confirms previous suggestions that the ‘Out of India’ hypothesis best explains the origin of Southeast Asian Heterometrinae, given their sister-group relationship with the African Pandininae, and the absence of Heterometrinae or Pandininae between the Middle East and the Indian subcontinent. However, prior to the present study, the pattern and timing of dispersal to Southeast Asia, including the question as to whether this involved a single colonization event, were unknown. As noted above, the timing and manner in which India collided with Eurasia, have been intensely debated. The analyses presented here are consistent with the model which proposes that India could have been connected to Southeast Asia as early as 57 Ma^26,28^ and gradually moved northwards until its final collision with Eurasia 35 Ma, and demonstrate that Heterometrinae dispersed into Southeast Asia on three independent occasions.

The earliest dispersal event occurred within *Heterometrus*. As India moved northward, it collided with an intra-oceanic island arc, the Dazhuqu Arc, ca. 55 Ma, at a time when its northern edge was subaerially exposed and close to Sumatra^26–28^. At around 56 Ma, according to the biogeographical analysis, one lineage of *Heterometrus*, the ancestor of *H. glaucus*, dispersed and diversified into Sundaland, just as the northern edge of Greater India and its island arc were approaching Southeast Asia, representing the first dispersal event within Heterometrinae (Fig. 4). At 44 Ma, when eastern India was adjacent to Sumatra^28^, a second dispersal event occurred in another genus when the ancestor of *Javanimetrus* dispersed from the Indian subcontinent to Sundaland (Fig. 4). The third dispersal event again involved *Heterometrus*, when the ancestor of the clade comprising all species of the genus except *H. glaucus* dispersed from the Indian subcontinent to Indochina and Sundaland ca. 37 Ma (Fig. 4). According to the models of Ali and Aitchison^27^ and Acton^28^, India was adjacent to Indochina around this time and this also constitutes the final collision event between India and Eurasia.

After the final dispersal event, ca. 37 Ma, *Heterometrus* appears to have followed a northwest to southeast pattern of dispersal as the species from Indochina are more basal in the phylogeny than those from Sundaland and the Philippines*.* The number of lineages also rose rapidly between 56 and 37 Ma, suggesting Asian scorpionids were diversifying into the new habitats encountered in Southeast Asia. This supports other studies which suggested accelerated biotic interchange for plants, vertebrates and various arthropods between Asia and India since the Eocene^98^. These findings are also congruent with a biogeographical analysis of rhacophorid frogs, which reconstructed an initial dispersal event into Southeast Asia, occurring 57 Ma, and several other dispersal events, occurring later^19^.

### Scorpionids disperse and diversify northwards across India

The phylogeny suggests the ancestors of *Chersonesometrus*, *Deccanometrus* and *Gigantometrus*, widespread across the interior of present-day India, dispersed across the subcontinent after the colonization of Southeast Asia. The collision of India with Eurasia resulted in the uplift of the Tibetan Plateau and the closure of the Paratethys Sea which, in turn, caused the shift from a zonal to monsoon climate on the Indian subcontinent, in the Miocene^25,99^. Although the precise timing of the onset of the Indian monsoons is debated, the effects of a monsoon climate were evident, with India significantly drier, by 8 Ma^98^. The seasonally drier climate changed the flora and fauna by reducing or eliminating the tropical evergreen forests of the lowlands and the moist mixed deciduous-pine forests of the highlands, allowing C_4_ plants, typical of savanna-grasslands, to dominate^100^. Fossil vertebrates from this time suggest a shift from forest-dwelling mammals to herbivores associated with open savanna^100,101^.

**Figure 5:**
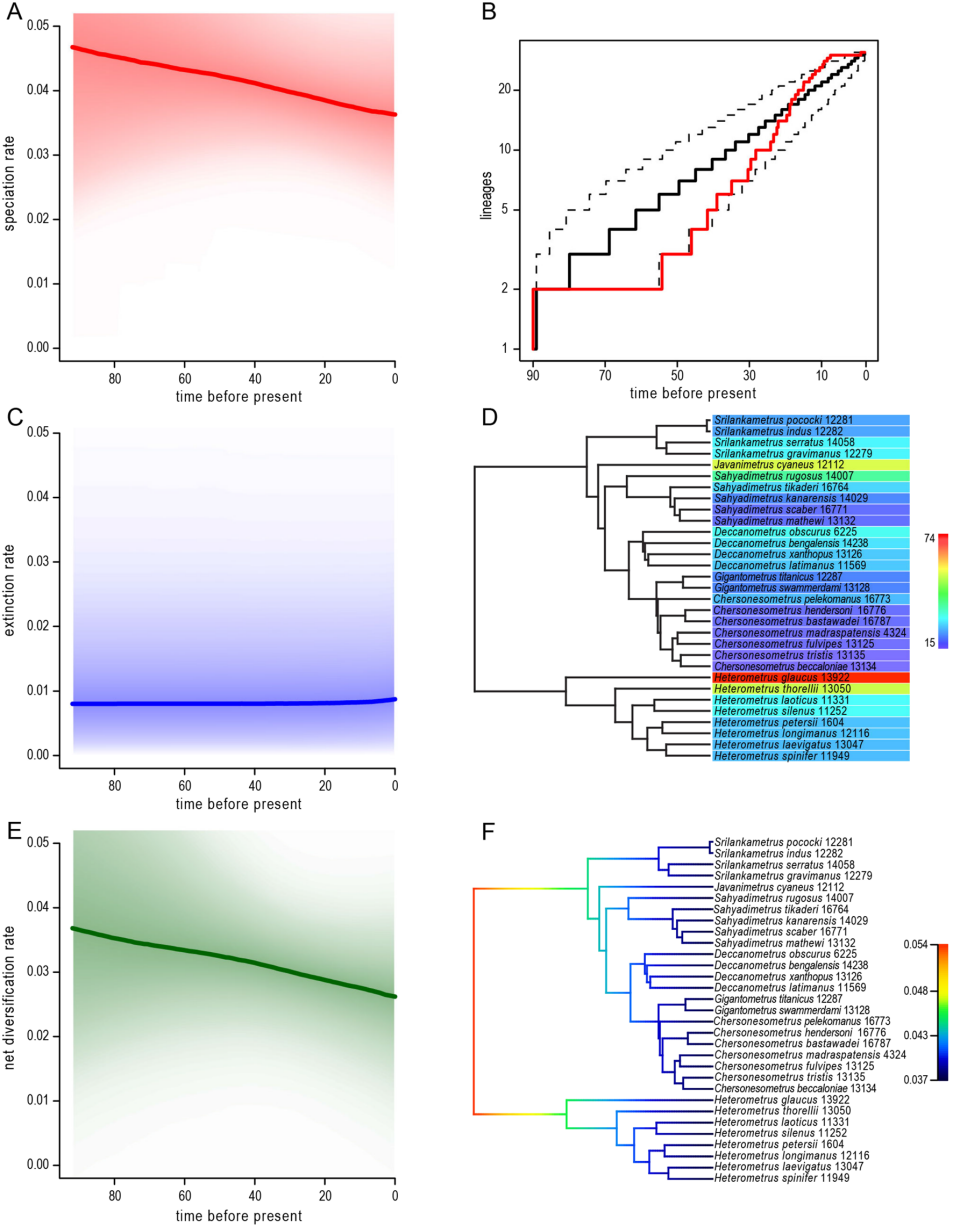
Diversification and evolutionary distinctiveness of the Asian forest scorpions (Scorpionidae: Heterometrinae): (**A**) Speciation rate. (**B**) Lineage through time plot. (**C**) Extinction rate. (**D**) Evolutionary distinctiveness per species. (**E**) Net diversification rate. (**F**) Phylorate plot indicating speciation rates among lineages.

Environmental changes occurring across the Indian subcontinent during the Miocene also allowed Indian scorpionids to take advantage of new habitats, resulting in the diversification of *Chersonesometrus*, *Deccanometrus* and *Gigantometrus*. Dispersal into more open, arid habitats was associated with ecomorphological adaptations. For example, *Deccanometrus latimanus* (Pocock, 1894) and *Deccanometrus xanthopus* (Pocock, 1897), which inhabit the savanna and scrub of Pakistan and India, respectively, exhibit reddish-brown coloration, robust pedipalps, metasoma and telson, and short, pale legs unlike the closely related species, *Deccanometrus bengalensis* (C.L. Koch, 1841) and *Deccanometrus phipsoni* (Pocock, 1893), which inhabit forests and exhibit uniformly blackish or dark brown coloration, more slender pedipalps, metasoma and telson, and longer legs^102^. According to the LTT plot (Fig. 5), the speciation rate reached a plateau by the beginning of the Middle Miocene (ca. 10 Ma) suggesting all new niches were occupied and diversification into new environments was complete.

### Alternative hypotheses

The analyses presented here, as well as evidence from other phylogenetic and biogeographical studies, refute Couzijn’s^23^ hypothesis for the origins, dispersal and diversification of Heterometrinae (Fig. 2). Couzijn^23^ suggested Heterometrinae diverged from their African relatives during the breakup of Gondwana and different lineages were transported to Asia on separate landmasses, the ancestors of *Gigantometrus*, *Chersonesometrus* and *Heterometrus* traveling on the Indian landmass, and the ancestor of *Javanimetrus* and *Srilankametrus* traveling on an Indochinese landmass. According to recent geological and biogeographical studies, however, the Indochinese landmass arrived at Southeast Asia in the Jurassic^103^, contradicting the timeline proposed by Couzijn^23^. Furthermore, Couzijn’s^23^ phenetic hypothesis of relationships among the genera recognized today, i.e., (*Gigantometrus* ((*Javanimetrus* + *Srilankametrus*) (*Chersonesometrus* + *Heterometrus*))), is contradicted by the phylogenetic reconstruction of Heterometrinae presented here and by Loria and Prendini^102^, i.e., (*Heterometrus* (*Srilankametrus* (*Javanimetrus* (*Sahyadrimetrus* (*Deccanometrus* (*Chersonesometrus* + *Gigantometrus*)))))).

Alternative hypotheses have been proposed to explain the origins and distribution patterns of other Gondwanan scorpions. Although Monod and Prendini^96^ used the Africa-India rift to explain the origins of an Indian clade of Hormuridae Laurie, 1896, the ‘Eurogondwana’ hypothesis^104^ was invoked to account for the distribution of an Indo-Pacific hormurid clade. According to this hypothesis, Hormuridae originated in Gondwana during the Paleozoic and the ancestors of this Indo-Pacific hormurid clade diverged in isolation from the ancestors of the African and South American hormurid genera, when the Apulian microplate rifted from Africa in the Early Cretaceous^105^. The Indo-Pacific hormurid clade subsequently arrived in Eurasia when the Apulian microplate collided into Europe and dispersed across the Palearctic region, eventually reaching Southeast Asia and Australasia. No hormurids presently occur in the Palearctic, hence Monod and Prendini^96^ suggested that the ‘icehouse’ climate in the Neogene and the ‘hothouse’ climate in the late Paleocene–early Eocene may have led to the extinction of these mostly tropical or subtropical, humidity-dependent scorpions.

The Eurogondwana hypothesis is a less likely explanation for the distribution patterns of Asian scorpionids. It implies the absence of scorpionids from the Palearctic, which is contradicted by the presence of *Scorpio* L., 1758 in northern Africa, the Arabian Peninsula and the Middle East (including part of Iran), suggesting scorpionids were able to survive severe climatic changes such as global warming and cooling. On the other hand, the ‘Out of India’ hypothesis is consistent with the geographical distribution of Pandininae, the sister group of Heterometrinae, which extends from Senegambia across western and central Africa to the Red Sea and the Gulf of Aden, and southward as far as the Zambezi River^5,67,95^. As discussed above, evidence exists that biotic exchange was still occurring between central Africa and India well into the Late Cretaceous and early Paleocene.

Sharma et al.^106^ produced a chronogram for scorpions, using crown and stem group ages for five arachnid taxa, including the scorpion family Buthidae C.L. Koch, 1837, estimated from fossil data, according to which superfamily Scorpionoidea began to diversify at the end of the Cretaceous, roughly 90 Ma. A biogeographical explanation for the early diversification of Scorpionoidea was not offered. Using five molecular markers and phylogenomic dating, Santibáñez et al.^107^ recovered the divergence between *Heterometrus* and *Pandinus* at the end of the Jurassic (145 Ma), but later^108^ suggested this divergence occurred in the Late Cenozoic (ca. 20 Ma). Whereas a Jurassic divergence is plausible, based on the present study and the geological literature, a Cenozoic divergence between *Heterometrus* and *Pandinus* is unlikely.

Clouse^109^ proposed a Cimmerian continent origin for the Southeast Asian harvestman family Stylocellidae Hansen and Sørensen, 1904. This scenario seems less plausible for Heterometrinae given that Cimmeria rifted from Gondwana ca. 290 Ma, and the dated tree presented here suggests that Heterometrinae diverged from the African Pandininae ca. 115 Ma.

## Conclusions

Understanding the biogeographical patterns of South and Southeast Asian taxa is confounded by the complex geological history of the region. Despite decades of research, there remains little consensus regarding how and when the Indian subcontinent collided with Eurasia or the events that unfolded during its northward journey. The analyses presented here provide a plausible reconstruction of the origins, dispersal and diversification patterns of another group of taxa, the Asian forest scorpions (Heterometrinae), which can be summarized into four major events. (1) Heterometrinae diverged from other Scorpionidae on the African continent after the Indian subcontinent became separated in the Cretaceous. (2) Environmental stresses during the KT mass extinction resulted in range contraction, restricting Heterometrinae to refugia in southern India (the Western Ghats) and Sri Lanka (the Central Highlands). (3) Heterometrinae dispersed to Southeast Asia three times during India’s collision with Eurasia, the first dispersal event occurring as the Indian subcontinent brushed up against the western side of Sumatra, and the other two events occurring as India moved closer to Eurasia. (4) Indian Heterometrinae, confined to southern India and Sri Lanka during the KT mass extinction, recolonized the Deccan Plateau and northern India, diversifying into new, more arid habitats after environmental conditions stabilized.

## Methods

### Taxon sampling

An extensive reconstruction of the phylogeny of Asian forest scorpions, based on 186 morphological characters and ca. 4200 DNA nucleotides from three mitochondrial and two nuclear gene loci for 132 terminals (Supplementary Figure) is published elsewhere^102^. The dataset was reduced to one terminal per species for the biogeographical analyses presented here, resulting in 35 terminals, representing all seven genera and 31 (76%) species of Heterometrinae, and an outgroup comprising exemplar species of three genera, representing each of the other subfamilies of Scorpionidae, from Africa and the Middle East (Supplementary Table 1): *Pandinus imperator* (C. L. Koch, 1841), representing Pandininae; *Scorpio fuscus* (Ehrenberg, 1829), representing Scorpioninae Latreille, 1802; and *Opistophthalmus capensis* (Herbst, 1800), representing Opistophthalminae Rossi, 2016. The tree was rooted on *Nebo hierichonticus* (Simon, 1872), representing Nebinae Kraepelin, 1905, the basal clade of Diplocentridae Karsch, 1880, the putative sister group of Scorpionidae^5,64^.

### DNA extraction and sequencing

Muscle tissue was dissected from the legs of specimens preserved in 95% ethanol, and DNA isolated using the DNEasy Blood and Tissue Extraction Kit (Qiagen, Hilden, Germany). Three mitochondrial gene loci, Cytochrome *c* Oxidase Subunit I (COI), 16S rDNA (16S) and 12S rDNA (12S), and two nuclear gene loci, 18S rDNA (18S) and 28S rDNA (28S), were sequenced. Previous research demonstrated this combination of loci was informative for resolving relationships at deep to shallow levels in scorpion phylogeny^5,110–113^. The COI and 18S loci were sequenced in two and three fragments, respectively, whereas all other loci were sequenced in single fragments^102^. Each locus was sequenced in both the forward and reverse directions. Sequence lengths were calculated using the gc_calculator.py script in Biopython^114^.

### DNA sequence alignment

Sequences were aligned using Mafft v. 7.429^115,116^ by auto-aligning with the ‘leavegappyregion’ option selected. The L-INS-I method, an iterative refinement method which uses a local pairwise alignment with an affine gap cost, was selected as the best alignment for all loci^117^. The aligned sequences were 1761 base-pairs (bp) in length for 18S, 515 bp for 28S, 341 bp for 12S, 493 bp for 16S, and 1078 bp for COI, summing to a total of 4188 bp for the concatenated dataset (Table 1). Gaps representing insertion/deletion events were present in the aligned 12S (17 gaps), 16S (12 gaps) and 28S (1 gap). Average percent A, C, G, T content was calculated for the aligned loci (Table 1) using MEGA v. 7^118^.

**Table 1:** Statistics for nuclear loci, 18S rDNA (18S) and 28S rDNA (28S), and mitochondrial loci, 12S rDNA (12S), 16S rDNA (16S) and Cytochrome *c* Oxidase subunit I (COI) in divergence time estimation analysis of Asian forest scorpions (Scorpionidae: Heterometrinae): length (nucleotide base-pairs) of unaligned and aligned sequences; percent of each nucleotide; and model selected for BEAST analysis using the Bayesian Information Criterion (BIC).

Locus	Unaligned	Aligned	BIC	A	C	G	T
18S	1761	1761	K80	25.3	23.4	27.7	23.5
28S	514–515	515	K80 + I + G	21.9	26.0	32.2	19.9
12S	328–335	341	HKY + I + G	39.5	9.7	16.9	33.9
16S	481–487	493	HKY + I + G	32.7	13.0	18.5	35.8
COI	1078	1078	GTR + I + G	19.3	13.0	24.7	43.0

### Divergence time estimation

A divergence time estimation analysis was performed in BEAST v. 1.10.4^119^. No fossil data exist for Heterometrinae hence a relaxed lognormal molecular clock was used to date the phylogeny with the following parameters in BEAUTi v. 1.10.4^119^. The diplocentrid, *N. hierichonticus*, was used as outgroup and a clade comprising Heterometrinae and *Pandinus* was constrained to be monophyletic based on evidence that the clade including *Pandinus* is the sister group of Heterometrinae^5,64^. Relationships among the ingroup were constrained to match the larger phylogenetic analysis of Heterometrinae^102^ (Supplementary Figure). The dataset was partitioned by loci with molecular clocks and substitution models unlinked across all partitions. An uncorrelated relaxed molecular clock was applied for each partition and models for individual loci selected using the Bayesian Information Criterion (BIC) in JModeltest v. 2^120^ on the CIPRES Science Gateway (Table 1)^121^. Trees were linked across all partitions and a birth–death tree prior implemented. Clock rates for the mitochondrial COI and 16S loci have been published for scorpion taxa from families Buthidae, Vaejovidae Thorell, 1876 and Bothriuridae Simon, 1880^122–131^. A normal clock rate prior was applied to the COI and 16S loci with the following settings: μ = 0.007, σ = 0.00146 for COI and μ = 0.005, σ = 0.00270 for 16S, such that 95% of the normal distribution included minimum and maximum values of the COI and 16S loci in scorpions^131^. Rates for all other loci were estimated using uniform priors with the following constraints: 12S, *ucld.min* = 0.002 and *ulcd.max* = 0.5; 18S and 28S, *ucld.min* = 0.0001 and *ulcd.max* = 0.01^127^. Two independent Bayesian analyses were conducted with the following settings: *mcmc*; *ngen* = 500,000,000; *print frequency* = 10,000; *sample frequency* = 10,000. Effective sample sizes for all parameters were above 200 and convergence between independent runs was assessed using Tracer v. 1.7.1^132^. Log Combiner v. 1.10.4 was used to combine tree files from the independent runs with burnin set to 25%. After burnin samples were removed, Tree Annotator v. 1.10.4 was used to compile a 50% majority rule consensus tree from the independent runs.

### Ancestral range estimation and phylogenetic diversity

Ancestral range reconstruction for the time-calibrated phylogenetic tree was implemented using the BioGeoBEARS v. 1.1.1 package^133–135^ in R v. 4.0.0^136^. Outgroups were excluded except for *Pandinus*, the sister group of Heterometrinae, using the *drop.tip* function from the R package ape^147^ and the tree was forced to be ultrametric using *force.ultrametic* from the R package phytools^148^. Six biogeographical areas were defined based on previous studies^60,137,138^: (A) Africa; (B) Western Ghats and Sri Lanka; (C) Greater Indian Subcontinent; (D) Indochina; (E) Sundaland; (F) Philippines (Fig. 3). The Isthmus of Kra was designated as the boundary between Indochina and Sundaland^137^. Each terminal taxon was scored as present or absent in each area. The maximum number of areas a species can occupy was assumed to be four. The analysis was time-stratified into five periods based on geological events^27^: 0–35 Ma, 35–45 Ma, 45–57 Ma, 57–68 Ma, 68–120 Ma (Supplementary Tables 2–4). Areas allowed and areas adjacent matrices (Supplementary Tables 3, 4) were included as well as a dispersal multiplier matrix with a probability of 1 for adjacent areas, 0.5 for areas with intermediate connections, and 1e−07 for areas with unlikely connection due to separation by large geographical distance or an ocean barrier (Supplementary Table 2). The models Dispersal-Extinction-Cladogenesis, DIVALIKE, and BAYAREALIKE were compared, and an additional parameter (*j*), which allows a founder speciation event, tested for each (Table 3). Statistical analyses were used to determine the likelihood of the dataset given each model and identify the most appropriate model for the dataset. Faith’s^139^ phylogenetic diversity (PD) index was applied to all ingroup areas (Table 4) using ten thousand randomizations of the ‘trial swap’ null model^140^ with the R package picante^141^. The evolutionary distinctiveness (ED) for each species was also calculated using the ‘fair proportions’^142^ and ‘equal splits’^143^ methods in picante.

**Table 2:** Ancestral areas of each genus of Asian forest scorpions (Scorpionidae: Heterometrinae) recovered by DEC + *j* analysis, with oldest and youngest divergence dates (millions of years) within each genus recovered by divergence time estimation analysis.

Genus	Ancestral area (code)	Oldest	Youngest
*Chersonesometrus*	Greater Indian Subcontinent (C)	21	10
*Deccanometrus*	Greater Indian Subcontinent (C)	26	24
*Gigantometrus*	Western Ghats and Sri Lanka (B)	10	–
*Heterometrus*	Greater Indian Subcontinent (C)	56	17
*Javanimetrus*	Greater Indian Subcontinent (C)	–	–
*Sahyadrimetrus*	Western Ghats and Sri Lanka (B)	32	12
*Srilankametrus*	Western Ghats and Sri Lanka (B)	21	1

**Table 3:** Statistics for models tested (DEC, DIVALIKE and BAY AREALIKE) including an additional founder effect (*j*) parameter in ancestral range estimation of the Asian forest scorpions (Scorpionidae: Heterometrinae): likelihoods (lnL), number of parameters in each model, dispersal (*d*), extinction (*e*), founder effect (*j*), Akaike Information Criterion (AIC), and corrected Akaike Information Criterion (AICc).

Model	lnL	Parameters	*d*	*e*	*j*	AIC	AICc
DEC	− 57.66	2	0.0041	0.0031	0	119.3	119.7
DEC + *j*	− 53.45	3	0.0026	0.0013	0.042	112.9	113.8
DIVALIKE	− 57.59	2	0.0052	0.0026	0	119.2	119.6
DIVALIKE + *j*	− 53.62	3	0.0029	0.0013	0.044	113.2	114.1
BAYAREALIKE	− 66.33	2	0.0042	0.0133	0	136.7	137.1
BAYAREALIKE + *j*	− 57.12	3	0.0023	0.0015	0.06	120.2	121.1

**Table 4:** Phylogenetic diversity (PD) of Asian forest scorpions (Scorpionidae: Heterometrinae) in six biogeographical regions (A–F): number of taxa per area (*n*); observed phylogenetic diversity (PD obs.); mean PD in null areas (PD rand.); standardized effect size of PD (*z*); probability (*p*).

Area (code)	*n*	PD obs.	PD rand.	*z*	*p*
Africa (A)	1	–	–	–	–
Western Ghats and Sri Lanka (B)	12	458.381	464.042	− 0.188	0.414
Greater Indian Subcontinent (C)	11	296.626	437.783	− 4.539	0.001
Indochina (D)	5	236.738	267.994	− 0.888	0.253
Sundaland (E)	6	192.383	301.735	− 3.256	0.002
Philippines (F)	2	140.024	146.636	− 0.251	0.507

### Diversification analyses

Outgroups were removed from the divergence time tree and the tree forced to be ultrametric as described above. A series of analyses were performed on the ingroup tree, to understand diversification patterns within Heterometrinae, using the R package laser v. 2.4-1^144^. The Akaike Information Criterion (AIC) was calculated to determine the best fitting model for the dataset. The command *fitdAICrc* was applied for the pure birth, birth–death, exponential density-dependent (DDX), linear density-dependent (DDL) and two-rate Yule models (Table 5). The commands *fitSPVAR* and *fitEXVAR* were applied for the variable speciation (SPVAR) and variable extinction (EXVAR) models^145^. Net diversification rates were calculated for a low extinction rate (ε = 0) and a high extinction rate (ε = 0.9), assuming low (*n* = 5) and high (*n* = 50) numbers of missing species, respectively. The gamma statistic, which assumes complete sampling, was calculated to determine whether the diversification rate is constant over time, using the *gamStat* (γ)^146^ function in the R package ape^147^. A Monte Carlo constant rates test, conducted using the function *mccrTest*, was also used to assess whether the observed gamma rate was affected by incomplete sampling, assuming ten different clade sizes (Table 6). A lineage-through-time (LTT) plot was generated, using the R package phytools^148^, to visualize lineage diversification in Heterometrinae, with 10,000 trees generated under a pure birth (Yule) model for comparison. Speciation rates, extinction rates, net diversification rates, and number of rate shifts were calculated by running Bayesian Analysis of Macroevolutionary Mixtures (BAMM) v. 2.5.0^149^ with the settings *nchains* = 4, *ngen* = 10,000,000 and initial priors selected using BAMMtools v. 2.1.6^150^ in R. The statistical significance of rate shifts was calculated using Bayes factors^151^. Results were visualized using BAMMtools.

**Table 5:** Statistical output of models tested in diversification analysis of Asian forest scorpions (Scorpionidae: Heterometrinae): extinction fraction (a), carrying capacity (K), speciation rate change (k), magnitude of rate-change (x), extinction rate change (z), speciation rate (λ).

Model	r1	r2	Model Parameters	LH	AIC	ΔAIC
**Rate-constant and variable rate models**						
pure birth	0.03378		107.1794 (a)	− 52.59	107.179	9.905
birth–death	0.03378		0 (a)	− 52.59	109.179	11.905
Yule 2-rate	0.04943	0.00641	37.67773 (st)	− 45.64	97.274	0
DDX	0.05376		0.17544 (x), 108.4224 (a)	− 52.21	108.422	11.148
DDL	0.07095		37.67773 (K), 103.2620 (a)	− 49.64	103.262	5.988
**Variable speciation/extinction models**						
SPVAR			0.04498 (λ), 0.00393 (k)	− 52.56	111.112	13.838
EXVAR			0.34234 (λ), 1.00755 (z)	− 52.66	111.319	14.045

**Table 6:** Statistical output of Monte Carlo Constant Rates Test using observed gamma value (γ = − 2.08) to determine effects of missing taxa on declining diversification rate in Asian forest scorpions (Scorpionidae: Heterometrinae).

Clade size	Missing taxa (%)	Critical value	*p*
41	10 (24)	− 2.020	0.044
42	11 (26)	− 2.039	0.453
43	12 (28)	− 2.103	0.052
44	13 (30)	− 2.125	0.056
48	17 (35)	− 2.222	0.066
52	21 (40)	− 2.356	0.087
62	31 (50)	− 2.570	0.135
78	47 (60)	− 2.909	0.241
103	72 (70)	− 3.280	0.396
124	93 (75)	− 3.492	0.508

## Supplementary information

Supplementary Information 1.
