# Supplementary material for: Out of India, thrice: diversification of Asian forest scorpions reveals three colonizations of Southeast Asia
Source: Sci Rep. 2020 Dec 18;10:22301. doi: 10.1038/s41598-020-78183-8 (PMC7749168; doi:10.1038/s41598-020-78183-8)

SUPPLEMENTARY INFORMATION

Out of India, Thrice: Diversification of Asian Forest Scorpions Reveals Three Colonizations of Southeast Asia

Stephanie F. Loria[1,2*] and Lorenzo Prendini[2]

[1]Richard Gilder Graduate School, American Museum of Natural History, Central Park West at 79th St., New York, NY 10024-5192, U.S.A.
[2]Scorpion Systematics Research Group, Division of Invertebrate Zoology, American Museum of Natural History, Central Park West at 79th St., New York, NY 10024-5192, U.S.A.

Contents

Supplementary Table 1. GenBank accession codes for DNA sequences of the 18S rDNA (18S), 28S rDNA (28S), 12S rDNA (12S), 16S rDNA (16S), and Cytochrome *c* Oxidase Subunit I (COI) loci used for phylogenetic analysis of the Asian forest scorpions (Scorpionidae: Heterometrinae). Tissue samples deposited in the Ambrose Monell Collection for Molecular and Microbial Research (AMCC) at the American Museum of Natural History, New York.

Supplementary Table 2. Dispersal multiplier matrix in the BIOGEOBEARS biogeographical analysis of Asian forest scorpions (Scorpionidae: Heterometrinae) indicating the probability for dispersal events between areas across five time periods. Dispersal events categorized as follows: 1, highest dispersal probability; 0.5, intermediate dispersal probability; and 0.0000001, little to no dispersal. Letters correspond to the areas defined in the analysis: (A) Africa, (B) Western Ghats and Sri Lanka, (C) Greater Indian Subcontinent, (D) Sundaland, (E) Indochina and (F) Philippines (Fig. 3).

Supplementary Table 3. Areas adjacent matrix in the BIOGEOBEARS biogeographical analysis of Asian forest scorpions (Scorpionidae: Heterometrinae) indicating adjacent (1) and nonadjacent (0) areas across five time periods. Letters correspond to areas defined in the analysis: (A) Africa, (B) Western Ghats and Sri Lanka, (C) Greater Indian Subcontinent, (D) Sundaland, (E) Indochina and (F) Philippines (Fig. 3).

Supplementary Table 4. Areas allowed matrix in the BIOGEOBEARS biogeographical analysis of Asian forest scorpions (Scorpionidae: Heterometrinae) indicating existing (1) and non-existing (0) areas across five time periods. Letters correspond to areas defined in the analysis: (A) Africa, (B) Western Ghats and Sri Lanka, (C) Greater Indian Subcontinent, (D) Sundaland, (E) Indochina and (F) Philippines (Fig. 3).

Supplementary Figure. Phylogeny of the Asian forest scorpions (Scorpionidae: Heterometrinae) obtained by Maximum Likelihood analysis of 186 morphological characters and ca. 4200 DNA nucleotides from two nuclear and three mitochondrial gene loci for 132 terminals (Loria and Prendini, 2020).

References

Loria, S. F. & Prendini, L. Burrowing into the forest: Phylogeny of the Asian forest scorpions (Scorpionidae: Heterometrinae) and the evolution of ecomorphotypes. *Cladistics.* https://doi.org/10.1111/cla.12434 (2020).

Supplementary Table 1. GenBank accession codes for DNA sequences of the 18S rDNA (18S), 28S rDNA (28S), 12S rDNA (12S), 16S rDNA (16S), and Cytochrome *c* Oxidase Subunit I (COI) loci used for phylogenetic analysis of the Asian forest scorpions (Scorpionidae: Heterometrinae). Tissue samples deposited in the Ambrose Monell Collection for Molecular and Microbial Research (AMCC) at the American Museum of Natural History, New York.

Family	Species	AMCC	Country	18S	28S	12S	16S	COI
Diplocentridae	*Nebo hierichonticus* (Simon, 1872)	LP 11138	Israel	MT417898	MT418125	MT417773	MT418351	MT418015
Scorpionidae	*Opistophthalmus capensis* (Herbst, 1800)	LP 1046	South Africa	MT417899	AY156532	AY156547	AY156562	AY156577
	Pandinus imperator (C.L. Koch, 1841)	LP 1601	Ghana	MT417900	AY156537	AY156552	AY156567	AY156582
	Scorpio fuscus (Ehrenberg, 1829)	LP 1556	Israel	MT417901	AY156539	AY156554	AY156569	AY156584
	Chersonesometrus bastawadei Prendini & Loria, 2020	LP 16787	India	MT417903	MT418127	MT417775	MT418353	MT418017
	Chersonesometrus beccaloniae (Kovařík, 2004)	LP 13134	India	MT417905	MT418129	MT417777	MT418355	MT418019
	Chersonesometrus fulvipes (C.L. Koch, 1837)	LP 13125	India	MT417906	MT418130	MT417778	MT418356	MT418020
	Chersonesometrus hendersoni Prendini & Loria, 2020	LP 16776	India	MT417912	MT418136	MT417784	MT418362	MT418026
	Chersonesometrus madraspatensis (Pocock, 1900)	LP 4324	India	MT417916	MT418139	MT417787	MT418365	MT418029
	Chersonesometrus pelekomanus (Couzijn, 1981)	LP 16773	India	MT417917	MT418140	MT417788	MT418366	MT418030
	Chersonesometrus tristis (Henderson, 1919)	LP 13135	India	MT417919	MT418142	MT417790	MT418368	MT418032
	Deccanometrus bengalensis (C.L. Koch, 1841)	LP 14238	India	MT417922	MT418145	MT417793	MT418371	MT418035
	Deccanometrus latimanus (Pocock, 1894)	LP 11569	Pakistan	MT417924	MT418147	MT417795	MT418373	MT418037
	Deccanometrus obscurus (Couzijn, 1981)	LP 6225	India	MT417929	MT418152	MT417800	MT418378	MT418042
	Deccanometrus xanthopus (Pocock, 1897)	LP 13126	India	MT417930	MT418153	MT417801	MT418379	MT418043
	Gigantometrus swammerdami (Simon, 1872)	LP 13128	India	MT417933	MT418156	MT417804	MT418382	MT418046
	Gigantometrus titanicus (Couzijn, 1981)	LP 12287	Sri Lanka	MT417936	MT418158	MT417806	MT418384	MT418048
	Heterometrus glaucus (Thorell, 1876)	LP 13922	Indonesia	MT417938	MT418160	MT417808	MT418386	MT418050
	Heterometrus laevigatus (Thorell, 1876)	LP 13047	Thailand	MT417943	MT418165	MT417813	MT418391	MT418055
	Heterometrus laoticus Couzijn, 1981	LP 11331	Laos	MT417947	MT418169	MT417817	MT418395	MT418059
	Heterometrus longimanus (Herbst, 1800)	LP 12116	Malaysia	MT417956	MT418178	MT417826	MT418404	MT418068
	Heterometrus petersii (Thorell, 1876)	LP 1604	Singapore	MT417958	AY156529	AY156544	AY156559	AY156574
	Heterometrus silenus (Simon, 1884)	LP 11252	Vietnam	MT417962	MT418183	MT417831	MT418409	MT418073
	Heterometrus spinifer (Ehrenberg, 1828)	LP 11949	Malaysia	MT417969	MT418189	MT417837	MT418415	MT418079
	Heterometrus thorellii (Pocock, 1892)	LP 13050	Myanmar	MT417974	MT418194	MT417842	MT418420	MT418084
	Javanimetrus cyaneus (C.L. Koch, 1836)	LP 12112	Indonesia	MT417981	MT418201	MT417849	MT418427	MT418091
	Sahyadrimetrus kanarensis (Pocock, 1900)	LP 14029	India	MT417988	MT418208	MT417856	MT418434	MT418098
	Sahyadrimetrus mathewi Prendini & Loria, 2020	LP 13132	India	MT417995	MT418215	MT417863	MT418441	MT418105
	Sahyadrimetrus rugosus (Couzijn, 1981)	LP 14007	India	MT417998	MT418218	MT417866	MT418444	MT418108
	Sahyadrimetrus scaber (Thorell, 1876)	LP 16771	India	MT418003	MT418223	MT417871	MT418449	MT418113
	Sahyadrimetrus tikaderi Prendini & Loria, 2020	LP 16764	India	MT418006	MT418226	MT417874	MT418452	MT418116
	Srilankametrus gravimanus (Pocock, 1894)	LP 12279	Sri Lanka	MT418009	MT418229	MT417877	MT418455	MT418119
	Srilankametrus indus (DeGeer, 1778)	LP 12282	Sri Lanka	MT418012	MT418232	MT417880	MT418458	MT418122
	Srilankametrus pococki Prendini & Loria, 2020	LP 12281	Sri Lanka	MT418011	MT418231	MT417879	MT418457	MT418121
	Srilankametrus serratus (Pocock, 1900)	LP 14058	Sri Lanka	MT418014	MT418234	MT417882	MT418460	MT418124

Supplementary Table 2. Dispersal multiplier matrix in the BIOGEOBEARS biogeographical analysis of Asian forest scorpions (Scorpionidae: Heterometrinae) indicating the probability for dispersal events between areas across five time periods. Dispersal events categorized as follows: 1, highest dispersal probability; 0.5, intermediate dispersal probability; and 0.0000001, little to no dispersal. Letters correspond to the areas defined in the analysis: (A) Africa, (B) Western Ghats and Sri Lanka, (C) Greater Indian Subcontinent, (D) Sundaland, (E) Indochina and (F) Philippines (Fig. 3).

Time Period 1	0–35 Ma					
	A	B	C	D	E	F
A	1	0.0000001	0.0000001	0.0000001	0.0000001	0.0000001
B	0.0000001	1	1	1	1	0.0000001
C	0.0000001	1	1	1	1	0.0000001
D	0.0000001	1	1	1	1	1
E	0.0000001	1	1	1	1	1
F	0.0000001	0.0000001	0.0000001	1	1	1

Time Period 2	35–45 Ma					
	A	B	C	D	E	F
A	1	0.0000001	0.0000001	0.0000001	0.0000001	0.0000001
B	0.0000001	1	1	1	1	0.0000001
C	0.0000001	1	1	1	1	0.0000001
D	0.0000001	1	1	1	1	0.0000001
E	0.0000001	1	1	1	1	1
F	0.0000001	0.0000001	0.0000001	0.0000001	1	1

Time Period 3	45–57 Ma					
	A	B	C	D	E	F
A	1	0.0000001	0.0000001	0.0000001	0.0000001	0.0000001
B	0.0000001	1	1	0.0000001	0.5	0.0000001
C	0.0000001	1	1	0.0000001	0.5	0.0000001
D	0.0000001	0.0000001	0.0000001	1	1	0.0000001
E	0.0000001	0.5	0.5	1	1	0.0000001
F	0.0000001	0.0000001	0.0000001	0.0000001	0.0000001	1

Time Period 4	57–68 Ma					
	A	B	C	D	E	F
A	1	0.0000001	0.0000001	0.0000001	0.0000001	0.0000001
B	0.0000001	1	1	0.0000001	0.0000001	0.0000001
C	0.0000001	1	1	0.0000001	0.0000001	0.0000001
D	0.0000001	0.0000001	0.0000001	1	1	0.0000001
E	0.0000001	0.0000001	0.0000001	1	1	0.0000001
F	0.0000001	0.0000001	0.0000001	0.0000001	0.0000001	1

Time Period 5	68–120 Ma					
	A	B	C	D	E	F
A	1	0.5	0.5	0.0000001	0.0000001	0.0000001
B	0.5	1	1	0.0000001	0.0000001	0.0000001
C	0.5	1	1	0.0000001	0.0000001	0.0000001
D	0.0000001	0.0000001	0.0000001	1	1	0.0000001
E	0.0000001	0.0000001	0.0000001	1	1	0.0000001
F	0.0000001	0.0000001	0.0000001	0.0000001	0.0000001	1

Supplementary Table 3. Areas adjacent matrix in the BIOGEOBEARS biogeographical analysis of Asian forest scorpions (Scorpionidae: Heterometrinae) indicating adjacent (1) and nonadjacent (0) areas across five time periods. Letters correspond to areas defined in the analysis: (A) Africa, (B) Western Ghats and Sri Lanka, (C) Greater Indian Subcontinent, (D) Sundaland, (E) Indochina and (F) Philippines (Fig. 3).

Time Period 1 0–35 Ma

	A	B	C	D	E	F
A	1	0	0	0	0	0
B	0	1	1	1	1	0
C	0	1	1	1	1	0
D	0	1	1	1	1	1
E	0	1	1	1	1	1
F	0	0	0	1	1	1

Time Period 2 35–45 Ma

	A	B	C	D	E	F
A	1	0	0	0	0	0
B	0	1	1	1	1	0
C	0	1	1	1	1	0
D	0	1	1	1	1	0
E	0	1	1	1	1	1
F	0	0	0	0	1	1

Time Period 3 45–57 Ma

	A	B	C	D	E	F
A	1	0	0	0	0	0
B	0	1	1	0	1	0
C	0	1	1	0	1	0
D	0	0	0	1	1	0
E	0	1	1	1	1	0
F	0	0	0	0	0	1

Time Period 4 57–68 Ma

	A	B	C	D	E	F
A	1	0	0	0	0	0
B	0	1	1	0	0	0
C	0	1	1	0	0	0
D	0	0	0	1	1	0
E	0	0	0	1	1	0
F	0	0	0	0	0	1

Time Period 5 68–120 Ma

	A	B	C	D	E	F
A	1	1	1	0	0	0
B	1	1	1	0	0	0
C	1	1	1	0	0	0
D	0	0	0	1	1	0
E	0	0	0	1	1	0
F	0	0	0	0	0	1

Supplementary Table 4. Areas allowed matrix in the BIOGEOBEARS biogeographical analysis of Asian forest scorpions (Scorpionidae: Heterometrinae) indicating existing (1) and non-existing (0) areas across five time periods. Letters correspond to areas defined in the analysis: (A) Africa, (B) Western Ghats and Sri Lanka, (C) Greater Indian Subcontinent, (D) Sundaland, (E) Indochina and (F) Philippines (Fig. 3).

Time Period 1 0–35 Ma

	A	B	C	D	E	F
A	1	1	1	1	1	1
B	1	1	1	1	1	1
C	1	1	1	1	1	1
D	1	1	1	1	1	1
E	1	1	1	1	1	1
F	1	1	1	1	1	1

Time Period 2 35–45 Ma

	A	B	C	D	E	F
A	1	1	1	1	1	1
B	1	1	1	1	1	1
C	1	1	1	1	1	1
D	1	1	1	1	1	1
E	1	1	1	1	1	1
F	1	1	1	1	1	1

Time Period 3 45–57 Ma

	A	B	C	D	E	F
A	1	1	1	1	1	1
B	1	1	1	1	1	1
C	1	1	1	1	1	1
D	1	1	1	1	1	1
E	1	1	1	1	1	1
F	1	1	1	1	1	1

Time Period 4 57–68 Ma

	A	B	C	D	E	F
A	1	1	1	1	1	0
B	1	1	1	1	1	0
C	1	1	1	1	1	0
D	1	1	1	1	1	0
E	1	1	1	1	1	0
F	0	0	0	0	0	0

Time Period 5 68–120 Ma

	A	B	C	D	E	F
A	1	1	1	1	1	0
B	1	1	1	1	1	0
C	1	1	1	1	1	0
D	1	1	1	1	1	0
E	1	1	1	1	1	0
F	0	0	0	0	0	0

Supplementary Figure. Phylogeny of the Asian forest scorpions (Scorpionidae: Heterometrinae) obtained by Maximum Likelihood analysis of 186 morphological characters and ca. 4200 DNA nucleotides from two nuclear and three mitochondrial gene loci for 132 terminals (Loria and Prendini, 2020).

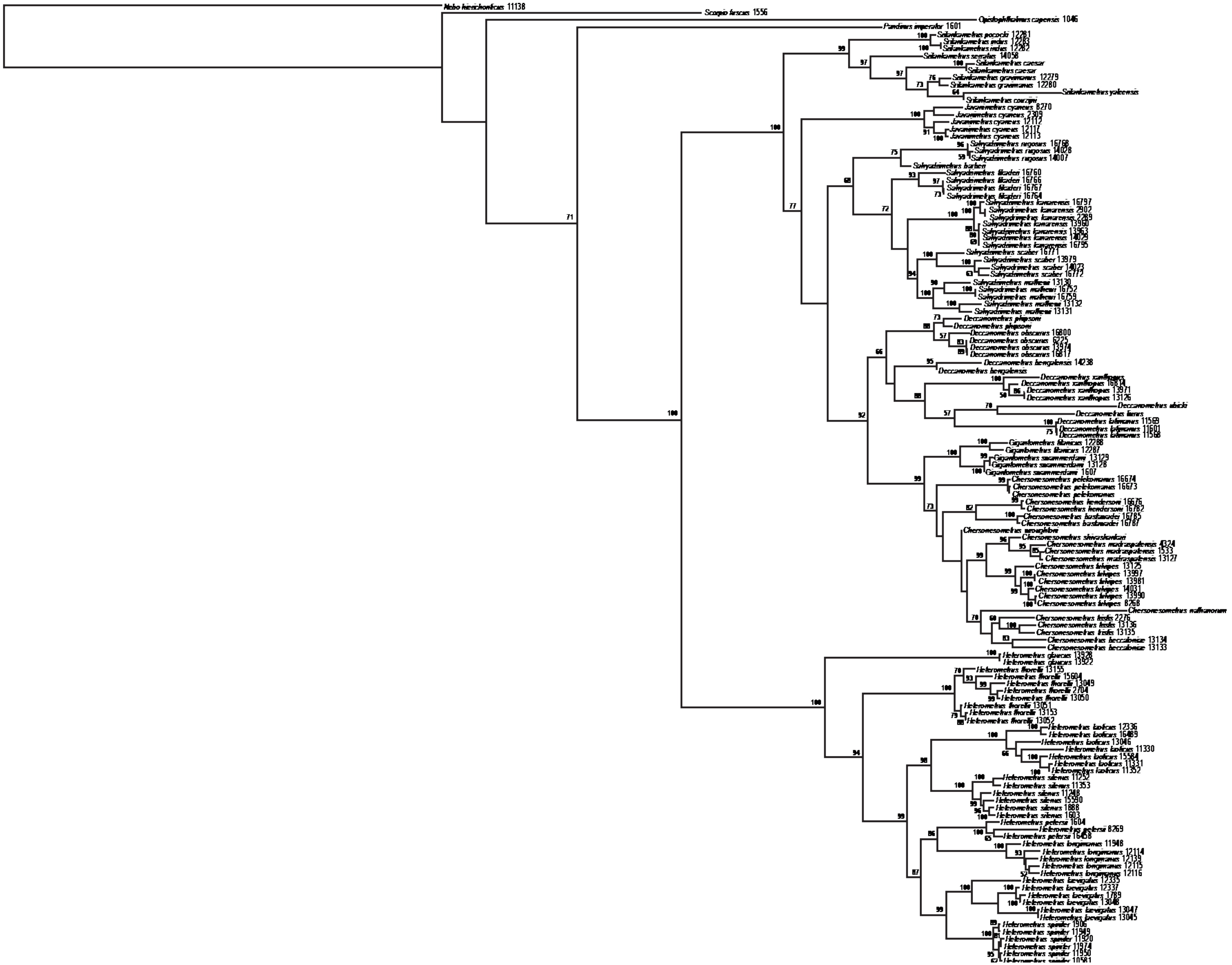

Supplement: Supplementary file 1 — Supplementary Information 1. [file 41598_2020_78183_MOESM1_ESM.pdf]
